# Fatores de risco nas neurocirurgias: um estudo de coorte no norte do Brasil*

**DOI:** 10.15649/cuidarte.2154

**Published:** 2022-10-17

**Authors:** Laís Xavier de Araújo, Priscilla Perez da Silva Pereira, Josimeire Cantanhede de Deus, Daniela Oliveira Pontes, Adriana Tavares Hang, Jeanne Lúcia Gadelha Freitas, Valéria Moreira da Silva, Karla de Paula Paiva, Caren Juliana Moura de Souza, Carla Vanessa Suaris Meireles, Mariana Delfino Rodrigues, Daniella Thamara da Silva Tavares, Marcela Miranda Sanches Rosa

**Affiliations:** 1 Universidade Federal de Rondonia (UNIR), Porto Velho, Brasil. Email: lais.xavier.777@gmail.com Universidade Federal de Rondônia Universidade Federal de Rondonia Porto Velho Brazil lais.xavier.777@gmail.com; 2 Universidade Federal de Rondonia (UNIR), Porto Velho, Brasil. Email: priperez@unir.br Universidade Federal de Rondônia Universidade Federal de Rondonia Porto Velho Brazil priperez@unir.br; 3 Universidade Federal de Rondonia (UNIR), Porto Velho, Brasil. Email: joosiwatson@gmail.com Universidade Federal de Rondônia Universidade Federal de Rondonia Porto Velho Brazil joosiwatson@gmail.com; 4 Universidade Federal de Rondonia (UNIR), Porto Velho, Brasil. Email: danielaopontes@hotmail.com Universidade Federal de Rondônia Universidade Federal de Rondonia Porto Velho Brazil danielaopontes@hotmail.com; 5 Universidade Federal de Rondonia (UNIR), Porto Velho, Brasil. Email: adrianahang@unir.br Universidade Federal de Rondônia Universidade Federal de Rondonia Porto Velho Brazil adrianahang@unir.br; 6 Universidade Federal de Rondonia (UNIR), Porto Velho, Brasil. Email: jeannegadelha@unir.br Universidade Federal de Rondônia Universidade Federal de Rondonia Porto Velho Brazil jeannegadelha@unir.br; 7 Universidade Federal de Rondonia (UNIR), Porto Velho, Brasil. Email: valeria.moreira@unir.br Universidade Federal de Rondônia Universidade Federal de Rondonia Porto Velho Brazil valeria.moreira@unir.br; 8 Universidade Federal de Rondonia (UNIR), Porto Velho, Brasil. Email: karlappaiva@yahoo.com.br Universidade Federal de Rondônia Universidade Federal de Rondonia Porto Velho Brazil karlappaiva@yahoo.com.br; 9 Universidade Federal de Rondonia (UNIR), Porto Velho, Brasil. Email: carenjms@outlook.com Universidade Federal de Rondônia Universidade Federal de Rondonia Porto Velho Brazil carenjms@outlook.com; 10 Universidade Federal de Rondonia (UNIR), Porto Velho, Brasil. Email: carlasuaris@yahoo.com.br Universidade Federal de Rondônia Universidade Federal de Rondonia Porto Velho Brazil carlasuaris@yahoo.com.br; 11 Universidade Federal de Rondonia (UNIR), Porto Velho, Brasil. Email: mdr.enf@gmail.com Universidade Federal de Rondônia Universidade Federal de Rondonia Porto Velho Brazil mdr.enf@gmail.com; 12 Universidade Federal de Rondonia (UNIR), Porto Velho, Brasil. Email: daniellathamara.enf@hotmail.com Universidade Federal de Rondônia Universidade Federal de Rondonia Porto Velho Brazil daniellathamara.enf@hotmail.com; 13 Universidade Federal de Rondonia (UNIR), Porto Velho, Brasil. Email: marcelamsanches@hotmail.com Universidade Federal de Rondônia Universidade Federal de Rondonia Porto Velho Brazil marcelamsanches@hotmail.com

**Keywords:** Infecgóes relacionadas a assistencia em saúde, Estudos Longitudinais, Neurocirurgia, Infections related to health care, Longitudinal Studies, Neurosurgery, Infecciones relacionadas con la atención médica, Estudios Longitudinales, Neurocirugía

## Abstract

**Introdujo:**

Pacientes neurocirúrgicos apresentam elevado risco de complicates locais e sistemicas que podem aumentar o tempo de internado e o risco de morte. Este estudo tem como objetivo avaliar a incidencia de infectes relacionadas a assistencia a saúde e os fatores de risco associados em pacientes submetidos as neurocirurgias.

**Materiais e Métodos:**

Estudo de coorte prospectiva, realizado em um Hospital de grande porte do estado de Rondonia, no período de 2018 a 2019, incluindo 36 pacientes.

**Resultados:**

A incidencia de infectes relacionada a assistencia a saúde foi 19,4 a cada 100 pacientes (IC95%: 8,19 - 36,02). Ter utilizado sonda nasoenteral aumentou em 6,5 vezes o risco de IRAS (IC 95%: 1,26 - 33,5), a ventilagáo mecánica aumentou 5,52 vezes o risco (IC95%: 1,23 - 24,6), a presenta de traqueostomia aumentou seis vezes (IC95%: 1,34 - 26,8) e realizagáo de exame invasivo aumentou o risco em 6,79 para ter infecto (IC95%: 1,31 - 35,05). Na análise ajustada as variáveis náo apresentaram significáncia estatística.

**Discussao:**

A incidencia de infectes foi maior do que em regióes com melhores condigóes socioeconómicas o que pode estar relacionado a menor adesáo de boas práticas na assistencia.

**Conclusao:**

Nas neurocirurgias além das infecgóes de sítio cirúrgico outras topografias também devem ser consideradas para investigagáo de infecgáo. O uso de dispositivos invasivos foi associado a ocorrencia de infecgóes relacionadas a assistencia a saúde, portanto as boas práticas no seu uso sáo essenciais no momento da indicagáo e uso destes dispositivos.

## Abstract (en)

**Introduction:**

Neurosurgical patients are at high risk of local and systemic complications that can increase the length of stay and the risk of death. This study aims to assess the incidence of healthcare-related infections and associated risk factors in patients undergoing neurosurgery.

**Materials and Methods:**

prospective cohort study, carried out in a large hospital in the state of Rondonia, in the period from 2018 to 2019, including 36 patients.

**Results:**

The incidence of healthcare-related infections was 19.4 per 100 patients (95% CI: 8,19 - 36,02). The use of nasoenteral tube increased the risk of HAI by 6.5 times (95% CI: 1,26 - 33,5), mechanical ventilation increased 5.52 times the risk (95% CI: 1,23 - 24,6), the presence of tracheostomy increased six times (95% CI: 1.34 - 26.8) and invasive examination increased the risk by 6.79 to have infection (95% CI: 1,31 - 35,05). In the adjusted analysis, the variables were not statistically significant.

**Discussion:**

The incidence of infections was higher than in regions with better socioeconomic conditions, which may be related to lower adherence to good care practices.

**Conclusion:**

in neurosurgeries, in addition to surgical site infections, other topographies should also be considered for investigation of infection. The use of invasive devices has been associated with the occurrence of healthcare-related infections, so good practices in their use are essential when referring and using these devices.

## Resumen

**Introducción:**

Los pacientes neuroquirúrgicos tienen un alto riesgo de complicaciones locales y sistémicas que pueden aumentar la duración de la estancia y el riesgo de muerte. Este estudio tiene como objetivo evaluar la incidencia de infecciones relacionadas con la atención médica y los factores de riesgo asociados en pacientes sometidos a neurocirugía.

**Materiales y métodos:**

estudio de cohorte prospectivo, realizado en un gran hospital del estado de Rondonia, en el período de 2018 a 2019, incluyendo 36 pacientes.

**Resultados:**

La incidencia de infecciones relacionadas con la asistencia sanitaria fue de 19,4 por 100 pacientes (IC del 95%: 8,19 - 36,02). Haber utilizado una sonda nasoenteral aumentó el riesgo de infecciones en 6,5 veces (IC 95%: 1,26 - 33,5), la ventilación mecánica aumentó 5,52 veces el riesgo (IC 95%: 1, 23 - 24,6), la presencia de traqueotomía aumentó seis veces (IC del 95%: 1,34 - 26,8) y el examen invasivo aumentó el riesgo en 6,79 de tener infecciones (IC del 95%: 1,31 - 35,05). En el análisis ajustado, las variables no fueron estadísticamente significativas.

**Discusión:**

La incidencia de infecciones fue mayor que en las regiones con mejores condiciones socioeconómicas, lo que puede estar relacionado con una menor adherencia a las buenas prácticas de atención.

**Conclusión:**

en neurocirugía, además de las infecciones del sitio quirúrgico, también deben considerarse otras topografías para la investigación de la infección. El uso de dispositivos invasivos se ha asociado con la aparición de infecciones relacionadas con la salud, por lo que las buenas prácticas en su uso son fundamentales a la hora de derivar y utilizar estos dispositivos.

## Introdujo

As Infecgóes Relacionadas a Assistencia a Saúde (IRAS) sao aquelas adquiridas após a admissáo do paciente no ambiente hospitalar e se manifestam no decorrer da internagáo ou após a alta. Essas infecgóes colocam em risco a Seguranza do Paciente (SP) e constituem-se como o Evento Adverso (EA) mais frequente nas instituyes hospitalares^1^.

Dentre as principais topografias das IRAS, a Infecgáo de Sitio Cirúrgico (ISC) está relacionada de modo direto aos procedimentos cirúrgicos. A ISC leva ao prolongamento do tempo de internado, além do aumento nos gastos devido ao seu tratamento^2^. Uma revisáo sistemática, incluindo 231 artigos publicados de 1995 a 2015, encontrou uma taxa combinada de ISC de 11,2 por 100 pacientes cirúrgicos (IC 95%: 9,7 - 12,8). Náo houve diferenga estatística nas taxas de ISC quando estratificadas por qualidade do estudo, grupos de idade dos pacientes, regióes geográficas, renda do país, critérios de definigáo de ISC, tipo de local ou ano de publicagáo. No entanto, houve diferengas estatísticas entre os estudos de acordo com o tipo de procedimentos e o número de pacientes por estudo^3^.

No Brasil, no ano de 2015, os dados sobre a incidencia de ISC em cirurgias variaram entre 1,4% a 38,8% a depender da instituigáo investigada e tipo de cirurgia^4^^-^^6^. Pacientes neurocirúrgicos apresentam elevado risco de complicagóes neurológicas e sistemicas, mesmo em procedimentos eletivos. As complicates sistemicas no pós-operatório de neurocirurgias eletivas incluem náuseas e vómitos, hipotensáo, desconforto respiratório e infecgáo do sítio cirúrgico. Em cirurgias náo eletivas, também estáo presentes dor e infecgóes nosocomiais. A taxa de mortalidade geral é de apenas 1% após neurocirurgia eletiva, em comparagáo com 29% após uma neurocirurgia de emergencia, frente as complicagóes pós-operatórias o risco de morte pode aumentar em ambos os grupos^6^.

As infecgóes em procedimentos neurológicos em geral sáo pouco frequentes quando comparadas com outros tipos de cirurgias, porém tem pior prognóstico, risco de sequelas e elevada letalidade^7^. Uma breve revisáo de literatura prévia indicou que é relevante a realizagáo de novos estudos que venham a esclarecer os principais tipos de infecgáo ocorridos nós pós-operatório, contribuindo para que os profissionais da área da saúde possam planejar agóes de diagnóstico precoce e boas práticas na assistencia aos pacientes submetidos as neurocirurgias^8^. Previamente a este estudo foram realizadas buscas nos sistemas de informagáo da instituigáo onde o estudo foi conduzido e nos bancos de dados da coordenagáo estadual de controle de infecgáo e seguranga do paciente do estado de Rondónia em busca de informagóes sobre a ocorrencia de infegóes nas neurocirurgias. Porém, náo foram encontrados registros que possibilitassem conhecer a realidade epidemiológica no que se refere aos procedimentos da neurologia.

No intuito de colaborar com o enriquecimento de informagóes acerca do tema e para subsidiar o planejamento e implantagáo de medidas de prevengáo por meio da adesáo de boas práticas, com a utilizagáo de regulamentos, diretrizes e compendios baseados em evidencias científicas, o presente estudo teve como objetivo avaliar a incidencia de infecgóes relacionadas a assistencia a saúde e os fatores de risco associados em pacientes submetidos as neurocirurgias.

## Materiais e Métodos

Foi conduzido um estudo de coorte, do tipo prospectivo, com pacientes submetidos a neurocirurgias em um Hospital de grande porte do estado de Rondónia, no município de Porto Velho, entre 2018 e 2019. A unidade hospitalar é de nível terciário e grande porte, possuía no período do estudo 590 leitos de média e alta complexidade e realizava em média 700 cirurgias ao mes.

Para definigáo do tamanho da amostra foi realizado um estudo piloto durante tres meses, onde 10 pacientes atenderam os critérios do estudo. Para o cálculo da amostra foi considerado nivel de confianza de 95%, poder do estudo 80%, razáo de um exposto para náo exposto, risco relativo encontrado no estudo piloto entre o tipo de cirurgia e a IRAS de 3,71. O cálculo da amostra para o estudo foi de 30 indivíduos e este valor foi acrescido com 10% para perdas totalizando 33 indivíduos.

Foram incluídos pacientes de ambos os sexos, com idade acima de 18 anos e que haviam realizado procedimentos cirúrgicos neurológicos. Foi considerado como paciente cirúrgico aqueles submetidos ao tratamento de traumatismos cranianos, traumas na coluna vertebral, doengas vasculares cerebrais como os aneurismas e a obstrugáo arterial, doengas vasculares da regiáo cervical que podem resultar em isquemia cerebral, da coluna e da medula, epilepsia, hérnias de disco, hidrocefalia, derrames e entre outras.

Foram excluidos tres individuos por apresentar internado inferior ou óbito até 48 horas após procedimento cirúrgico e com história prévia de infecgáo hospitalar confirmada e náo tratada durante a internado na unidade ou em outras unidades.

As informales sociodemográficas, condigóes clínicas e de notificado de infecgáo hospitalar foram coletadas do Sistema de Gerenciamento Hospitalar (Hospub) do Ministério da Saúde (prontuário eletronico) e prontuário físico por meio de questionário próprio elaborado pelos próprios autores. Após avaliagáo dos critérios de elegibilidade, eram coletadas as informagóes sociodemográficas e do procedimento cirúrgico. A coleta de dados sobre a internagáo ocorreu a cada 48 horas durante toda a internagáo do paciente.

A variável dependente foi a Infecgáo Relacionada a Assistencia a Saúde. Foram consideradas as quatro principais topografias das IRAS: Infecgáo de Sitio Cirúrgico (ISC), Infecgáo da Corrente Sanguinea (ICS), Infecgáo do Trato Urinário (ITU) e a Pneumonia associada a Ventilagáo Mecánica (PAV)9. As IRAS foram declaradas no prontuário pelo médico plantonista ou médico da comissáo de controle de infecgáo hospitalar (CCIH). A instituigáo segue os critérios estabelecidos pelo National Nosocomial Infections Surveillance (NNIS/CDC) traduzido e publicado no Brasil pela Agencia Nacional de Vigiláncia Sanitária (ANVISA)^9^.

As variáveis independentes foram organizadas em tres grupos, que se referem as caracteristicas demográficas, informagóes sobre o procedimento cirúrgico e internagáo. A base de dados tem acesso público em Mendeley Data^10^.

Características demográficas: local de moradia (capita ou interior); idade (<59 anos e > 60 anos); sexo (feminino e masculino); raga/cor da pele (branca e náo branca - preta, parda ou amarela); estado civil (com companheiro e sem companheiro); escolaridade (< 9 anos de estudo e > 9 anos de estudo).

Informagóes sobre o procedimento: tipo de cirurgia (limpa, potencialmente contaminada, contaminada e infectada); anestesia geral (sim ou náo); tempo de duragáo do procedimento (<120 ou >120 minutos); uso de implante (náo ou sim); antibiótico profilático (sim ou náo).

Informagóes sobre a internagáo: presenga de dispositivos (sim ou náo); total de dias em uso de dispositivos e número de trocas (acesso venoso central e/ou periférico, sonda nasoenteral, ventilagáo mecánica, traqueostomia, sonda vesical de demora e drenos); pós-operatório na unidade de terapia intensiva (sim ou náo); administragáo de hemoderivados (sim ou náo); parada cardiorrespiratória (sim ou náo); e realizagáo de exame invasivo (sim ou náo).

A incidencia foi calculada considerando no numerador o número de pacientes com IRAS, no denominador o total de pacientes submetidos a procedimentos cirúrgicos, considerando a taxa por 100 pacientes e foi calculado o respectivo intervalo de confianga a 95%.

Para avaliar as associagóes das variáveis com a infecgáo hospitalar foi realizada análise por meio de frequencia e medidas de tendencia central, bivariada por meio do teste qui-quadrado de Pearson e teste exato de Fisher para verificar a associagáo entre a variável dependente e as demais. Todas as variáveis com o teste de significáncia menor que 0,10 (p<0,10) foram submetidos a análise múltipla por meio da regressáo de Poisson utilizando-se a estratégia stepwise forward selection. As covariáveis foram testadas quanto a possível presenta de multicolinearidade - representada por correlates superiores a 0,80 e estas variáveis nao foram consideradas no modelo final para determinar o risco relativo e seu respectivo intervalo de confianza a 95%. A análise estatística foi realizada com auxilio do programa estatístico STATA® versao 16.0 (College Station, Texas, USA).

Este estudo faz parte do projeto matriz sobre "Infecgóes relacionadas a assisténcia a saúde no Estado de Rondonia: incidencia e fatores associados" e foi aprovado sob o parecer de n° 2.866.650 pelo Comité de Ética em Pesquisa da Universidade Federal de Rondonia. Foram respeitados os principios éticos para pesquisas envolvendo seres humanos de acordo com as normas estabelecidas pela resolugáo no 466/2012 do Conselho Nacional de Saúde do Brasil^11^.

## Resultados

Do total de 1.545 cirurgias realizadas no período do estudo, 39 foram neurológicas e 36 pacientes atendiam aos critérios de elegibilidade deste estudo. Sete pacientes tiveram diagnóstico de IRAS durante a internado - um com diagnóstico de ICS, um com ITU, um com ISC, um com PAV, um topografia náo definida, um com PAV e ITU e um com ICS e ISC. A taxa de incidéncia de IRAS entre os pacientes submetidos a neurocirurgias foi 19,4 a cada 100 pacientes (IC95%: 8,19 - 36,02).

A média de idade foi 58,25 anos (DP 14,74; mínimo 33 e máximo 91 anos). A maioria dos pacientes era do sexo feminino, possuía companheiro, ensino fundamental completo, autodeclarava-se náo branco e pouco mais da metade era do interior do estado. Nenhuma característica demográfica apresentou associagáo estatisticamente significativa as IRAS (Tabela 1).

**Tabela 1:** Informales demográficas dos pacientes submetidos a procedimento neurológico, Porto Velho/RO, 2018-2019 (n=36)

Variável	Sem IRAS n=29 n (%)	Com IRAS n=7 n (%)	Valor de ^p^
Local de moradia			0,40
Capital	15 (88,24)	2 (11,76)	
Interior	14 (73,68)	5 (26,32)	
Idade			0,99
Até 59 anos	15 (78,95)	4 (21,05)	
Acima de 60 anos	14 (82,35)	3 (17,65)	
Sexo			0,38
Feminino	18 (75,00)	6 (25,00)	
Masculino	11 (91,67)	1 (8,33)	
Raca/cor da pele*			0,99
Branca	6 (85,71)	1 (14,29)	
Outras*	15 (78,95)	4 (21,05)	
Estado civil*			0,21
Com companheiro	14 (70,00)	6 (30,00)	
Sem companheiro	11 (91,67)	1 (8,33)	
Escolaridade*			0,99
< 9 anos de estudo	7 (77,78)	2 (22,22)	
> 9 anos de estudo	19 (79,17)	5 (20,83)	

Quanto as informales sobre o procedimento cirúrgico, nenhuma cirurgia foi classificada como de emergencia. A maioria das cirurgias foi classificada como limpa, teve tempo de duragáo superior a 120 minutos, os pacientes receberam anestesia geral e pouco mais da metade fez uso de antibiótico profilático (Tabela 2). Quanto ao uso de implantes, em 27,78% dos procedimentos foram utilizadas sendo em 60% do tipo clip. Nenhuma variável relacionada ao procedimento apresentou associagáo estatisticamente significativa com as IRAS.

**Tabela 2:** Informales sobre o procedimento neurológico, Porto Velho/RO, 2018-2019 (n=36)

Variável	Sem IRAS n (%)	Com IRAS n (%)	Valor de ^p^
Tipo de cirurgia			0,57
Limpa	25 (78,13)	7 (21,88)	
Nao limpa	4 (100,00)	0 (0,00)	
Anestesia geral*			0,56
Nao	5 (100,00)	0 (0,00)	
Sim	24 (80,00)	6 (20,00)	
Tempo de duracao do procedimento*			0,62
< 120 minutos	5 (71,43)	2 (28,57)	
> 120 minutos	17 (80,95)	4 (19,05)	
Uso de implante			0,99
Nao	21 (80,77)	5 (19,23)	
Sim	8 (80,00)	2 (20,00)	
Em uso de antibiótico profilático no dia			0,10
do procedimento cirúrgico			
Nao	15 (93,75)	1 (6,25)	
Sim	14 (70,00)	6 (30,00)	

Após o procedimento neurológico, a maioria dos pacientes possuía acesso venoso, 75% utilizaram sonda vesical de demora e 52,78% utilizaram drenos. Quanto a administrado de dieta, 27,78% possuíam sonda nasoenteral. Do total de pacientes internados 19,44% utilizaram ventilado mecánica e 11,11% traqueostomia. Em relado a associagáo com as IRAS, o uso de sonda nasoenteral (p=0,01), ventilado mecánica (p=0,01), traqueostomia (p=0,01), uso de hemoderivado (p=0,01) e a realizado de exames invasivos (p=0,03) apresentaram associagáo estatisticamente significativa a IRAS. A variável - número de trocas de acesso venoso apresentou p=0,05 um valor limítrofe (Tabela 3).

**Tabela 3:** Informales sobre a internado dos pacientes submetidos a procedimento neurológico, Porto Velho/RO, 2018-2019 (n=36)

Variável	Sem IRAS n (%)	Com IRAS n (%)	Valor de ^p^
Acesso venoso central			0,15
Nao	9 (100,00)	0 (0,00)	
Sim	20 (74,07)	7 (25,93)	
Total de dias com acesso venoso central			0,20
Até 5 dias	9 (90,00)	1 (10,00)	
> 6 dias	11 (64,71)	6 (35,29)	
Número de trocas do acesso venoso*			0,05
Nenhuma	16 (80,00)	4 (20,00)	
Pelo menos uma vez	1 (25,00)	3 (75,00)	
Sonda nasoenteral			0,01
Nao	24 (92,31)	2 (7,69)	
Sim	5 (50,00)	5 (50,00)	
Ventilado mecánica			0,01
Nao	26 (89,66)	3 (10,34)	
Sim	3 (42,86)	4 (57,14)	
Total de dias em ventilado mecánica			0,47
Até 7 dias	2 (66,67)	1 (33,33)	
> 8 dias	1 (25,00)	4 (75,00)	
Traqueostomia			0,01
Nao	28 (87,50)	4 (12,50)	
Sim	1 (25,00)	3 (75,00)	
Uso de sonda vesical de demora			0,15
Nao	9 (100,00)	0 (0,00)	
Sim	20 (74,07)	7 (25,93)	
Total de dias com o dispositivo			0,08
Até 7 dias	15 (83,33)	3 (16,67)	
> 8 dias	4 (50,00)	4 (50,00)	
Drenos			0,09
Nao	16 (94,12)	1 (5,88)	
Sim	13 (68,42)	6 (31,58)	
Foi para a UTI após procedimento cirúrgico			0,30
Nao	7 (100,00)	0 (0,00)	
Sim	22 (75,86)	7 (24,14)	
Recebeu hemoderivados			0,01
Nao	28 (87,50)	4 (12,50)	
Sim	1 (25,00)	3 (75,00)	
Parada cardiorrespiratória			0,09
Nao	28 (84,85)	5 (15,15)	
Sim	1 (33,33)	2 (66,67)	
Realizou exame invasivo			0,03
Nao	29 (85,29)	5 (14,71)	
Sim	0 (0,00)	2 (100,00)	

Na análise de risco relativo bruto, ter utilizado sonda nasoenteral aumentou em 6,5 vezes o risco de IRAS (IC 95%: 1,26 - 33,5), a ventilagáo mecánica aumentou 5,52 vezes o risco (IC95%: 1,23 - 24,6), a presenta de traqueostomia aumentou seis vezes (IC95%: 1,34 - 26,8) e realizado de exame invasivo aumentou o risco em 6,79 para ter IRAS (IC95%: 1,31 - 35,05; Tabela 4). Na análise ajustada, as variáveis náo apresentaram significáncia estatística.

**Tabela 4:** Análise do risco relativo bruto, Porto Velho/RO, 2018-2019 (n=36)

Variável	RR (IC95%)	Valor de p
Características Sociodemográficas		
Local de moradia. Interior	2,23 (0,43 - 11,53)	0,33
Idade. Acima de 60 anos	0,83 (0,18 - 3,74)	0,81
Sexo. Masculino	0,33 (0,04 - 2,76)	0,30
Raca/cor da pele*. Outras	1,47 (0,16 - 13,18)	0,72
Estado civil*. Sem companheiro	0,27 (0,03 - 2,30)	0,23
Escolaridade. < 9 anos de estudo	0,93 (0,18 - 4,83)	0,93
Informales sobre o procedimento cirúrgico		
Tipo de cirurgia. Nao limpa	NC	
Anestesia geral. Sim	NC	
Tempo do procedimento. > 120 minutos	0,66 (0,12 - 3,64)	0,64
Uso de implante. Sim	1,04 (0,20 - 5,36)	0,96
Uso de antibiótico profilático no dia do procedimento. Nao	0,20 (0,02 - 1,73)	0,14
Informales sobre a internado		
Acesso venoso central. Sim	NC	
Total de dias com acesso venoso central. > 6 dias	3,52 (0,42 - 29,32)	0,24
Número de trocas do acesso venoso*. Nenhuma vez	3,75 (0,83 - 16,75)	0,08
Sonda nasoenteral. Sim	6,50 (1,26 - 33,50)	0,02
Ventilado mecánica. Sim	5,52 (1,23 - 24,68)	0,02
Total de dias em ventilado mecánica. > 3 dias	2,25 (0,23 - 21,63)	0,48
Traqueostomia. Sim	6,00 (1,34 - 26,81)	0,01
Uso de sonda vesical de demora. Sim	NC	
Total de dias com o dispositivo. > 8 dias	3,00 (0,67 - 13,40)	0,15
Drenos. Sim	NC	
Foi para a UTI após procedimento cirúrgico. Sim	NC	
Parada cardiorrespiratória. Sim	4,40 (0,85 - 22,68)	0,07
Realizou exame invasivo. Sim	6,79 (1,31 - 35,05)	0,02

NC - nao calculado, pois na tabela 2x2 continha opgóes com zero participantes. *Dados faltosos

Neste estudo, a média de internagáo após a cirurgia foi 12,86 (DP: 13,16, mínimo: 3, máximo 57). Apenas 8,33% dos pacientes foram transferidos para outros hospitais, 80,56% tiveram alta e 11,11% (quatro) evoluíram para óbito.

## Discussao

A taxa de incidencia de IRAS neste estudo foi 19,4 a cada 100 pacientes. Características sociodemográficas e da cirurgia náo apresentaram risco estatisticamente significante as IRAS. O uso de sonda nasoenteral, ventilado mecánica, presenta de traqueostomia e realizado de exame invasivo foram fatores de risco para IRAS na análise bruta, náo apresentando significáncia estatística na análise ajustada.

No estado de Rondonia, encontrou-se a predominancia de neurocirurgias no sexo feminino, média de idade de 58 anos e com nove ou mais anos de estudo. Uma pesquisa realizada no Servido de Atendimento Móvel de Urgencia e Emergencia no estado do Rio Grande do Norte com 73 participantes, encontrou pouco mais da metade das situagóes de emergencias neurológicas entre o sexo masculino (52,1%), pacientes com mais de 60 anos (58,9%) e com até nove anos de estudo (86%), portanto situagóes advindas principalmente de acidentes de transito ou decorrentes do envelhecimento^12^. Em Rondonia, os participantes da pesquisa foram pacientes de um hospital de cirurgias eletivas, ou seja, que possuíam condigóes de aguardar pelo procedimento cirúrgico. Procedimentos neurológicos de emergencia advindos dos acidentes de transito, por exemplo, sao tratados em outras instituigóes de saúde do estado.

O tipo de procedimento neurocirúrgico em si mesmo pode levar a maior probabilidade de ocorrencia de infecgao. Um estudo de revisao de literatura apontou que pacientes que recebem shunts apresentam infecgóes em 4 a 17% das vezes. Já a craniotomia a infecgao ocorre entre 5 a 8% e cirurgias na coluna espinhal 6%^13^. A taxa de incidencia para IRAS em pacientes submetidos a neurocirurgias em Rondonia foi 19,4/100 pacientes (IC95%: 8,19 - 36,02), valor superior ao encontrado em um estudo de coorte retrospectivo de tres anos, conduzido em um hospital universitário de um país Árabe, com 913 pacientes em tratamento neurológicos onde encontrou uma taxa de incidencia geral de IRAS de 11,9/100 pacientes, entre os quais 29% tiveram múltiplas infecgóes^14^. Outro estudo realizado na China, no período de maio de 2007 a abril de 2012, que incluiu pacientes submetidos as cirurgias cranianas identificou pneumonia no período pós-operatório em 0,5% a 4,1% dos pacientes^15^.

No Brasil, na capital do Amazonas, estado vizinho de Rondonia, a infecgao hospitalar entre pacientes submetidos a clipagem de aneurisma cerebral esteve presente em 21,8% dos pacientes^5^. Um estudo de coorte retrospectiva realizada em seis hospitais da capital do estado de Minas Gerais, por um período de dez anos, encontrou uma frequencia de 8,8% para infecgóes globais e 5,1% as infecgóes de sítio cirúrgico para cirurgias de craniotomia^16^. Em Sao Paulo, uma pesquisa do tipo prospectivo, conduzida em um hospital universitário, com a participagao de 85 pacientes adultos submetidos a neurocirurgia, no período de junho de 2012 a abril de 2013, encontrou a ocorrencia de ISC em 9,4% dos casos e os fatores de risco associados com a presenga de infecgao foram: tempo total de internagao, nao ser eutrófico, cirurgias de grande porte e ocorrencia de transfusao sanguínea^17^.

Percebe-se que os valores de IRAS encontrados nos dois estudos da regiao Norte, ambos em contextos piores condigóes socioeconomicas, sao bem superiores aos dos dois estudos do Sudeste. Este fato aponta para reflexóes sobre as diferengas existentes no acesso as tecnologias, implementagao de boas práticas na assistencia ao paciente por meio da cultura de seguranga do paciente, adogao de protocolos e disponibilidade de recursos humanos para implementar agóes de vigilancia e educagao permanente sobretudo no ambito de unidades hospitalares de maior porte e complexidade, a exemplo do local do presente estudo.

Porém, uma adequada estrutura física e implementagao de protocolos de boas práticas nao sao os únicos fatores que interferem na ocorrencia das infecgóes. Um fato importante a ser considerado é que o diagnóstico precoce de infecgóes no pós-operatório de neurocirurgias pode ser dificil. Muitas vezes os sintomas sao vagos, inespecíficos, podem existir antes da cirurgia ou pode ser atribuído diretamente ao diagnóstico neurológico primário subjacente. Da mesma forma, os pacientes podem ter um nível de consciencia rebaixada o que dificulta a queixa de sintomas e um detalhado exame neurológico. Exames laboratoriais de sangue também podem ser inespecíficos e os exames complementares como a coleta de liquor cefaloraquidiano por si só pode aumentar o risco de introduzir secundariamente infecgao^13^.

 Em Rondonia, encontrou-se que o uso de dispositivos como sonda nasogástrica ou nasoenteral, ventilado mecánica, traqueostomia e a historia de realizado de exames invasivos apresentaram-se como fatores de risco as IRAS. Um estudo conduzido na cidade de Manaus, incluindo 57 pacientes, encontrou um resultado que corrobora com nossos achados. Os autores apresentaram que o desenvolvimento de IRAS estava diretamente relacionado com procedimentos invasivos como sondagens e ventilado mecánica e nao com a intervengo neurocirúrgica^18^.

Uma revisáo sistemática com 54 estudos sobre fatores relacionados as infecgóes de neurocirurgias para tratamento de aneurismas encontrou que o sexo, história de uso de anticoagulante, tabagismo, hipertensáo, diabetes, insuficiencia cardíaca congestiva, local e calcificado do aneurisma foram identificados como fatores de risco intrinsecos ao paciente^19^. Outra revisao sistemática com 29 estudos sobre fatores de risco para infecgáo de sitio cirúrgico em craniotomia encontrou que a historia de infecgóes prévias, número de cirurgias, vazamento e drenagem de liquido cefalorraquidiano, tempo de duragáo da cirurgia, sexo, tipo de cirurgia traumática ou náo, técnica de tricotomia, uso de antibiotico profilático, acessos venosos, uso de dreno e implantes também foram fatores de risco para as infecgóes^20^.

Conhecendo os fatores de risco para a infecgáo nas neurocirurgias é importante trazer as principais remomendagóes para evitar a ocorrencia das mesmas. Entre elas estáo o controle dos níveis pressóricos e de glicemia, cessar o tabagismo, diminuigáo do tempo de cirurgia, adequado preparo da pele com clorexidina visando a eliminagáo de microrganismos, uso de antibiotoico profilático, tecnicas assépticas e suporte adequado de oxigenagáo^21^.

Este estudo aponta para a necessidade da qualificagáo dos processos de cuidado ao paciente das neurocirurgias principalmente no que se refere ao uso de dispositivos. Entretanto, os resultados encontrados precisam ser analisados considerando a pequena amostra quando comparado a outros estudos, apesar de terem sido incluídos todos os pacientes que atendiam os critérios de elegibilidade no período de um ano. Porém, os resultados seguiram tendencias de outros estudos maiores e pode ser útil para trazer um perfil situacional a ser considerado por outros hospitais da regiáo Norte com estrutura similar. Outra fraqueza do estudo foi em relagáo a fonte de dados, as informagóes disponibilizadas nos prontuários muitas vezes se apresentam com dados faltosos e informagóes incompletas, o que interferiu na análise de algumas variáveis. Porém, essa ausencia de informagáo de qualidade também é um dado importante, pois aponta para a necessidade de revisáo dos processos de trabalho no que se refere ao relato das informagóes, o que interfere diretamente no proprio diagnostico de IRAS.

## Conclusao

A taxa de incidencia de IRAS neste estudo foi 19,4 a cada 100 pacientes submetidos a cirurgias neurologicas. O uso de sonda nasoenteral, ventilagáo mecánica, presenga de traqueostomia e realizagáo de exame invasivo foram fatores de risco para IRAS na análise bruta, náo apresentando significáncia estatística na análise ajustada.

A gestáo do cuidado em saúde abrange aspectos múltiplos que envolvem desde o conhecimento dos profissionais, o nível e a complexidade das atividades assistenciais, até disponibilidade e a distribuigáo de recursos humanos e estrutura física. Para futuros estudos, sugere-se investigar a percepgáo dos profissionais quanto as boas práticas para evitar a ocorrencia de IRAS podendo assim realizar um diagnostico situacional que, juntamente com o perfil levantado neste estudo, possa subsidiar o planejamento de agóes em prol do controle de infecgóes na instituigáo.
